# Supplementary material for: There Is No Evidence That Inactivated COVID-19 Vaccines Increase Risks of Uveitis Flare
Source: Vaccines (Basel). 2022 Oct 8;10(10):1680. doi: 10.3390/vaccines10101680 (PMC9612251; doi:10.3390/vaccines10101680)
Supplement: Supplementary file 1 [file vaccines-10-01680-s001.zip › vaccines-1943775-supplementary.pdf]

Table S1. Detailed information for the 14 patients who had a uveitis flare within 60 days of vaccination.

No.	Age	Sex	Time interval between flare and vaccination	Etiology	History of uveitis before the flare	Flare type and presentations	Treatment and outcome after the flare
1	51	F	3 days after the third dose.<br>No flare after the first 2 doses.	VKH	8 years of pan-uveitis uveitis history, uveitis stable for around one year under treatment of prednisone, MTX and MMF.	Anterior uveitis; Blurred vision.	Flare controlled by topical steroids.
2	36	M	27 days after the third dose.<br>No flare after the first 2 doses.	Idiopathic	7 years of pan-uveitis uveitis history, with flare rate every one or two years. Patient was under no systemic treatment before this flare. Patient had been overworking and alcoholic after vaccination. Flare happened following a cold.	Anterior uveitis; Red eye and eye pain.	Flare controlled by topical steroids and rest.
3	38	M	33 days after the third dose.<br>No flare after the first 2 doses.	PSS	7 years of anterior uveitis history, flare frequency was every 2 months to 9 months. Patient was treated with ganciclovir.	Anterior uveitis; No complaint. Flare detected by a regular follow-up.	Flare controlled by topical steroids.
4	34	M	8 days after the second dose.<br>No flare after the first and third dose.	Idiopathic	7 years history of unilateral anterior uveitis without systemic treatment. Flare frequency was every 2 months to 6 months for the past year.	Anterior uveitis; Blurred vision and red eye.	Flare controlled by topical steroids.
5	54	M	20 days after the third dose. No flare after the first 2 doses.	Idiopathic	4 years history of pan-uveitis with frequent flares that often requires topical steroids and periocular triamcinolone injection. Under no systemic treatment around vaccination date.	Pan-uveitis; Blurred vision.	Flare treated with periocular triamcinolone injection 3 times during 4 months. After a short inflammatory quiescent period, patient underwent cataract surgery
6	32	M	30 days after the second dose. No flare after the first dose. The third dose not injected.	BDU	Pan-uveitis onset half a year before vaccination, had the first flare after the second dose vaccination. Patient was under no systemic treatment before the flare	Anterior uveitis; Blurred vision.	Flare controlled by topical steroids. Patient had frequent flares four months later and was diagnosed with BDU afterwards thus started TAC treatment.
7	39	M	22 days after the second dose. No flare after the first dose. The third dose not injected.	PSS	9 months history of PSS with frequent flares but no systemic treatment.	PSS flare; Eye pain ,increased intraocular pressure and presence of KP.	Flare controlled by topical steroids as always.
8	43	F	10 days after the second dose. No flare after the first dose. The third dose not injected.	Idiopathic	10 years history of anterior uveitis under no systemic treatment. Recent flare rate was 3 to 4 times per year.	Anterior uveitis; Red eye, eye pain and blurred vision.	Flare controlled by topical steroids as always.
9	35	M	22 days after the second dose. No flare after the first dose. The third dose not injected.	Idiopathic	3.5 years history of anterior uveitis, with 2 flares in total. The last flare was 16 months before vaccination.	Anterior uveitis; Red eye, eye pain.	Flare controlled by topical steroids and the patient was added with MTX due to positive HLA-B27 detected.
10	29	F	22 days after the second dose. No flare after the first dose. The third dose not injected.	Fuchs syndrome	8 months history of Fuchs uveitis, with 2 flares in total. Flare started soon after topical steroids cessation.	Anterior uveitis; Red eye, eye pain	Flare controlled by topical steroids but another flare followed up soon after topical steroids reduction thus MTX was added.
11	53	F	Had the first and second vaccination during persistent inflammatory status.<br>The third dose not injected.	VKH	1.5 years history of pan-uveitis. Had been treated with prednisone and intravitreous Orzudex 3 times due to uncontrolled and persistent inflammation.	Pan-uveitis; Red eye, eye pain and Blurred vision.	Refractory cases of VKH that could not be controlled. MTX and TAC were added after the first does of vaccination. Inflammation controlled around 3 months after treatment augmentation.

12	39	M	42 days after the second dose. No flares within 2 months after the first or the third dose.	BDU	5 years history of Behcet' s disease, with uveitis stable for at least one year without treatment.	Pan-uveitis; Blurred vision. Vasculitis and macular edema.	Patient started TAC and prednisone to avoid visual acuity damage in BDU. Inflammation was controlled with no additional flare during the 3 months follow-up period.
13	20	F	41 days after the first dose. No flare after the second dose. Third dose not injected.	Idiopathi c	4 years of pan-uveitis treated with prednisone, TAC and MMF. Flares occurred 1-2 times per year.	Anterior uveitis; Blurred vision.	Flare was controlled by topical steroids and had been inflammatory quiescent during the 8 months' follow-up period.
14	32	F	15 days after the second dose. No flares between the first and second dose or within 2 months of the third dose.	Idiopathi c	New onset of anterior uveitis 3 months before vaccination. Patient was under no topical or systemic treatment around the vaccination date.	Anterior uveitis; Red eye, eye pain.	Flare was controlled by topical steroids. Had flares every 3-5 months afterwards during the 11 months follow-up period.
